# Supplementary figures and images for: Differential requirement of NPHP1 for compartmentalized protein localization during photoreceptor outer segment development and maintenance
Source: PLoS One. 2021 May 7;16(5):e0246358. doi: 10.1371/journal.pone.0246358 (PMC8104407; doi:10.1371/journal.pone.0246358)

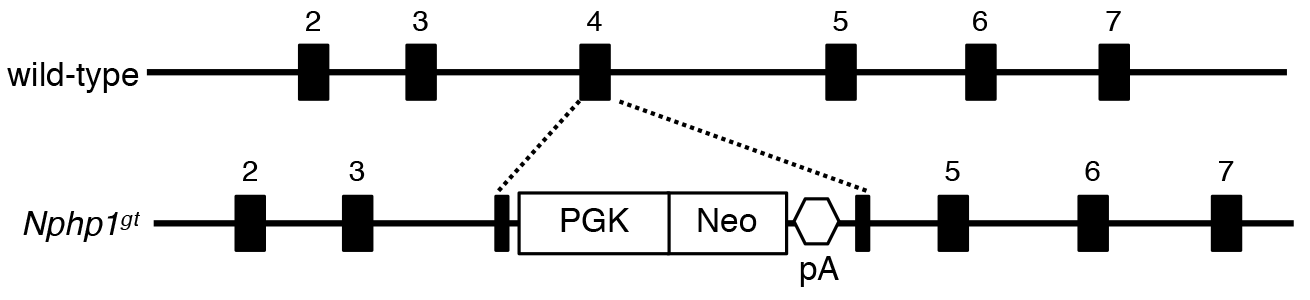

Supplement: S1 Fig — Black boxes represent Nphp1 exons. Exon numbers are described above the black boxes. The PGK-Neo gene-trap is inserted into Nphp1 exon 4. PGK: mouse phosphoglycerate kinase 1 promoter, Neo: neomycin resistance gene, pA: poly(A) signal. (TIF) [file pone.0246358.s001.tif]

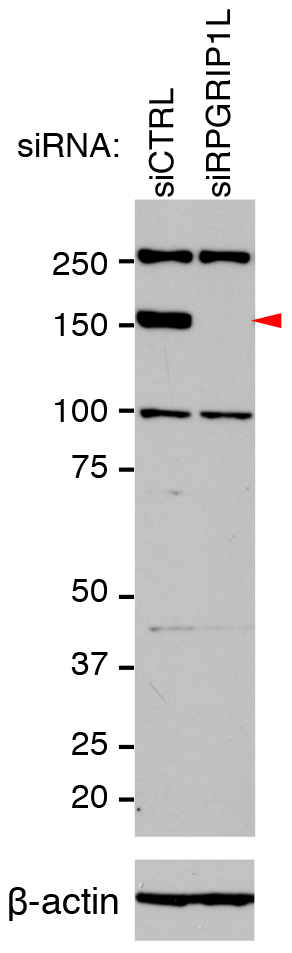

Supplement: S2 Fig — hTERT-RPE1 cells (ATCC #CRL-4000) were transfected with control (siCTRL; Horizon ON-TARGETplus Non-targeting Pool cat#: D-001810-10-05) or human RPGRIP1L siRNAs (siRPGRIP1L; Horizon ON-TARGETplus SMARTpool cat# L-022557-00-0005) using Lipofectamine RNAiMAX (Invitrogen), and cell lysates were loaded on an SDS-PAGE gel for immunoblotting with anti-RPGRIP1L antibodies. β-actin was used as a loading control. RPGRIP1L band was marked with a red arrowhead. (TIF) [file pone.0246358.s002.tif]

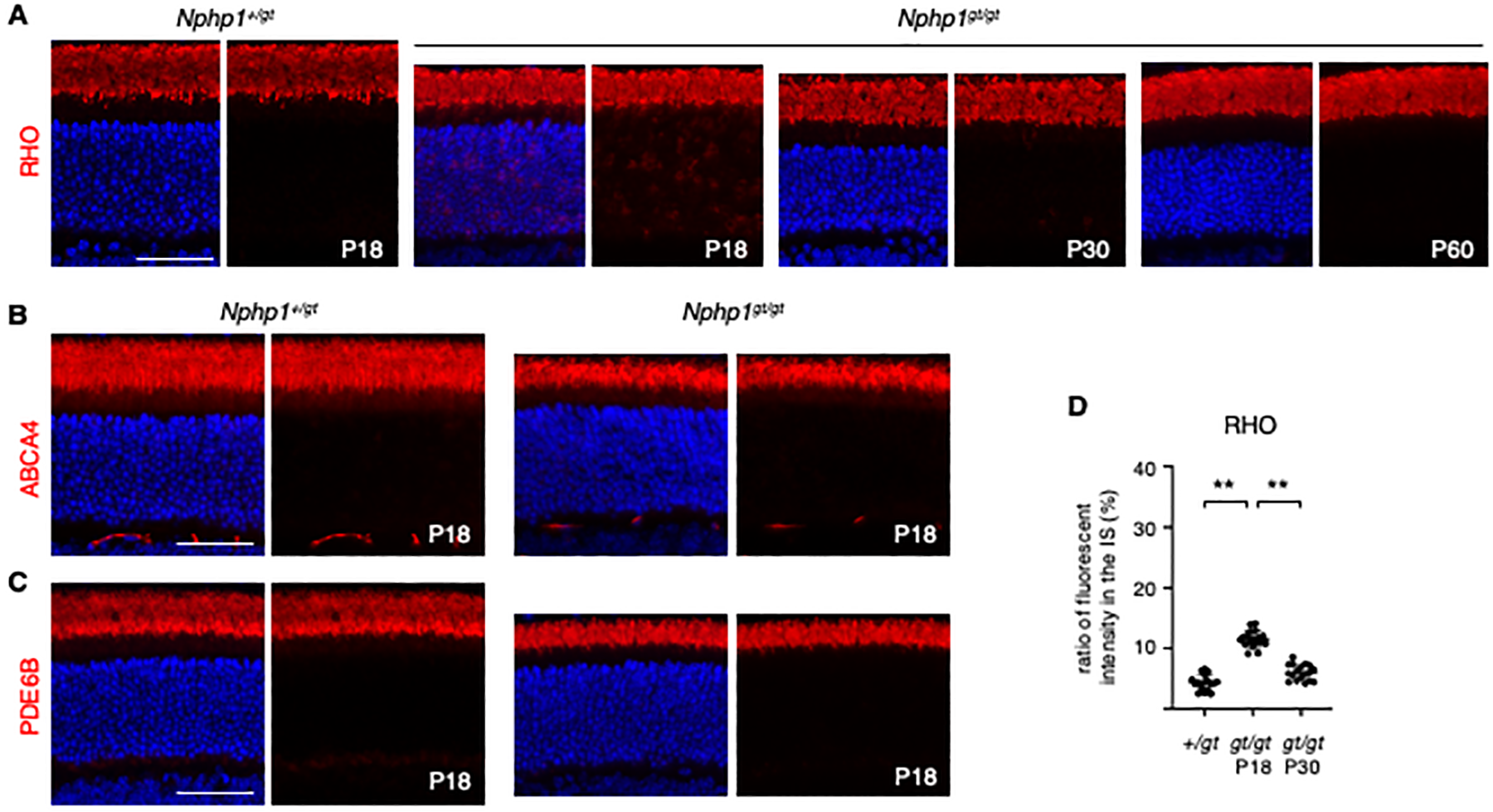

Supplement: S3 Fig — (A-C) Localization of RHO (A), ABCA4 (B), and PDE6B (C) in Nphp1gt/gt retinas. Retinal sections from Nphp1+/gt and Nphp1gt/gt mice were stained with RHO, ABCA4, and PDE6B antibodies (red). DAPI (blue) was used to counterstain the nuclei. Mild mislocalization of RHO was detected in Nphp1gt/gt retinas at P18. Localization of ABCA4 and PDE6B was not affected. Scale bar denotes 50 μm. (D) Quantification of RHO mislocalization to the IS. Depicted is the ratio of integrated fluorescence intensity in the IS relative to the photoreceptor cell layer. Mean ± SD (error bars) is marked by horizontal lines (n = 3 mice; 2 sections/mouse and 3 areas/section). Asterisks indicate statistical significance (one-way ANOVA followed by Tukey’s multiple comparison test; p < 0.01). (TIFF) [file pone.0246358.s003.tiff]

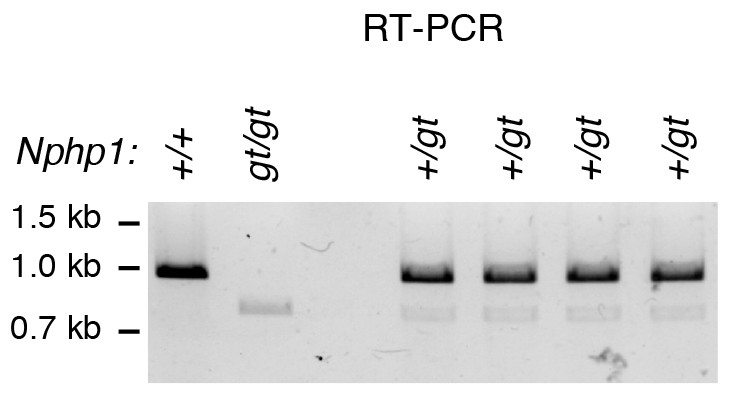

Supplement: S4 Fig — (TIF) [file pone.0246358.s004.tif]

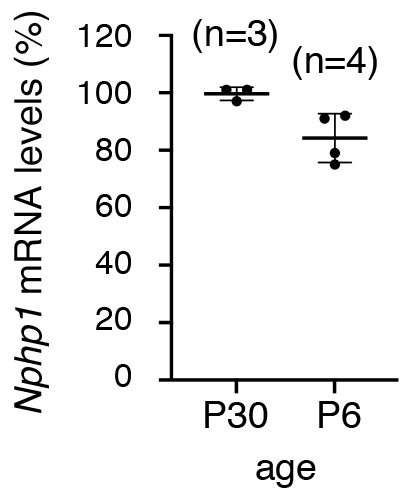

Supplement: S5 Fig — (TIF) [file pone.0246358.s005.tif]

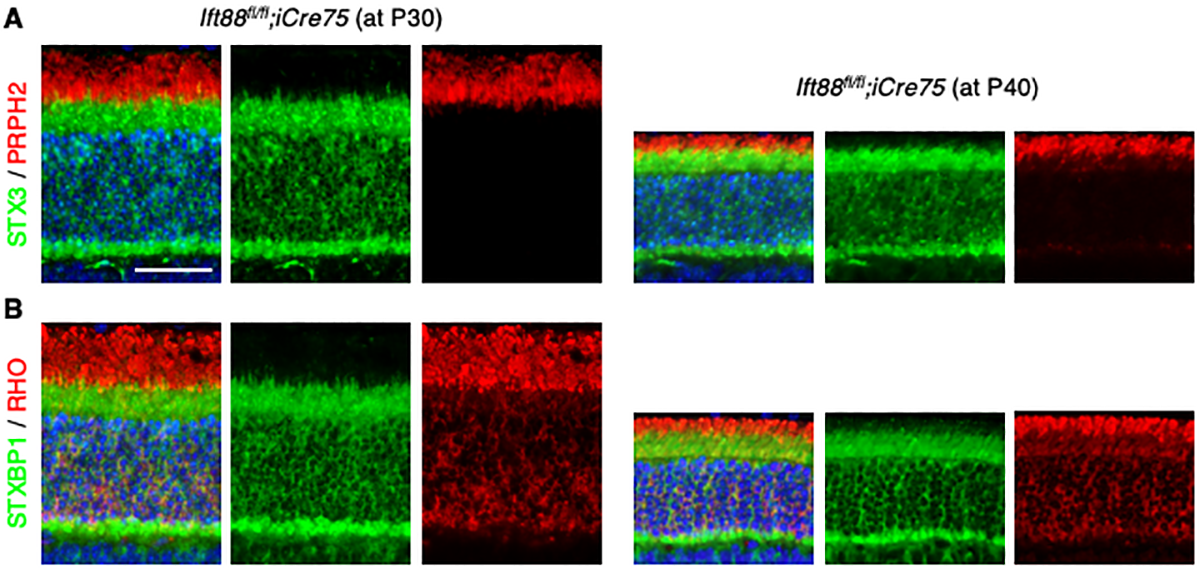

Supplement: S6 Fig — Retinal sections from 30-day and 40-day old Ift88fl/fl;iCre75 mice were stained with STX3 (A) and STXBP1 (B) antibodies (green). PRPH2 and RHO (red) were labeled as a marker of the OS. Merged images are shown on the left in each set. DAPI (blue) was used to counterstain the nuclei. Scale bar denotes 50 μm. (TIF) [file pone.0246358.s006.tif]
