# Supplementary material for: Differential requirement of NPHP1 for compartmentalized protein localization during photoreceptor outer segment development and maintenance
Source: PLoS One. 2021 May 7;16(5):e0246358. doi: 10.1371/journal.pone.0246358 (PMC8104407; doi:10.1371/journal.pone.0246358)
Supplement: S1 File — (PDF) [file pone.0246358.s007.pdf]

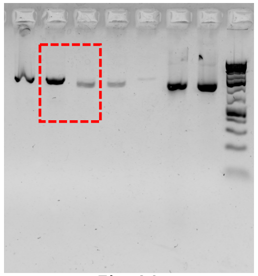

Fig 4A
RT-PCR (left)

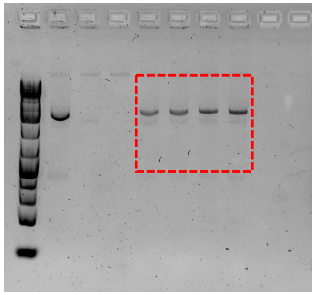

Fig 4A
RT-PCR (right)

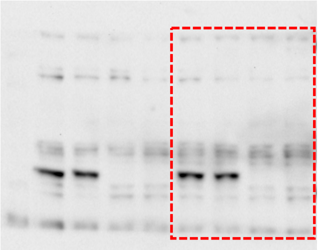

Fig 5A
NPHP1

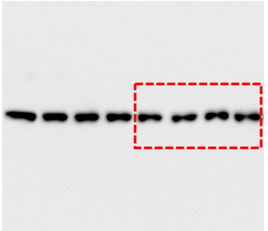

Fig 5A
β-actin

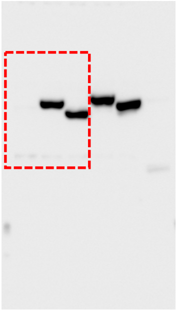

Fig 5B
HA

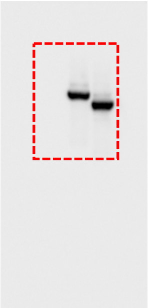

Fig 5B
NPHP1

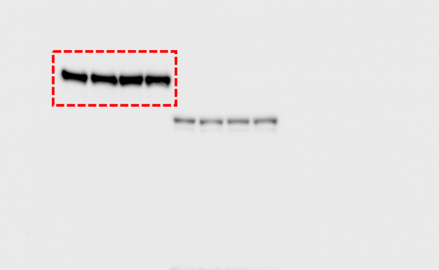

Fig 6A
GFP-NPHP1 (input)

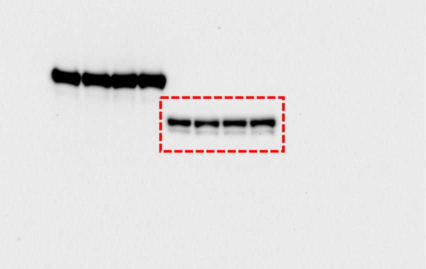

Fig 6B
GFP-NPHP5 (input)

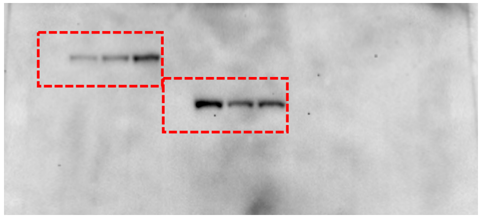

Fig 6A,B
GFP (IP)

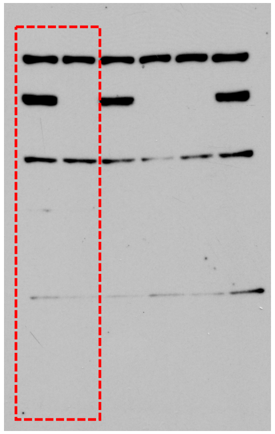

Supplementary Fig S2
(RPGRIP1L)

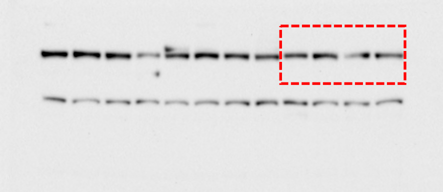

Fig 6C
RPGRIP1L (input)

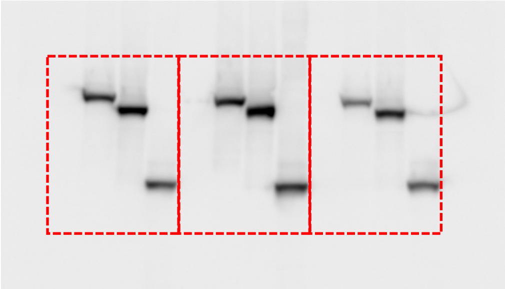

Fig 6A,B,C
FLAG (input)

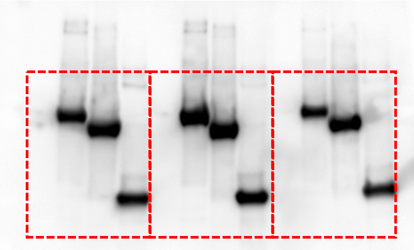

Fig 6A,B,C
FLAG (IP)

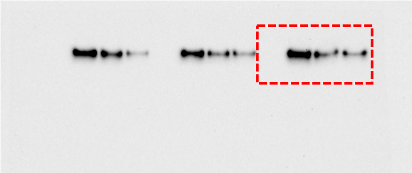

Fig 6C
RPGRIP1L (IP)

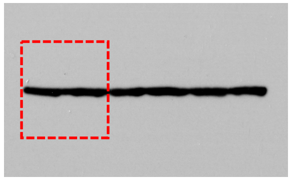

Supplementary Fig S2
β-actin

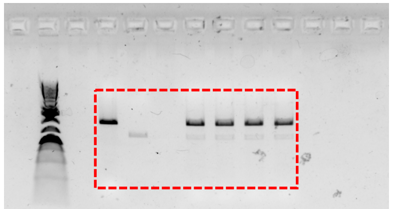

Supplementary Fig S4
